# Maintenance of approach velocity despite increasing internal load during repeated pole vault attempts

**DOI:** 10.1038/s41598-026-58645-1

**Published:** 2026-06-17

**Authors:** Hilal Kalkan, Savaş Akbaş, Serdar Orkun Pelvan, Hasan Birol Çotuk, Saif Al-Jumaili, Adil Deniz Duru, Frederico Branco, Ivan Miguel Pires

**Affiliations:** 1 Institute of Health Sciences, Marmara University, İstanbul, Turkey; 2 Faculty of Sport Sciences, Marmara University, İstanbul, Turkey; 3 Faculty of Science, Turkish-German University, İstanbul, Turkey; 4 Institute of Biophysics and Biomedical Engineering (IBEB), Faculty of Sciences, University of Lisbon, Lisbon, Portugal; 5 AquaValor—Centro de Valorização e Transferência de Tecnologia da Água, 5400-342 Chaves, Portugal; 6 Institute for Systems and Computer Engineering, Technology and Science (INESC TEC), 4200-465 Porto, Portugal; 7 School of Science and Technology, Universidade de Trás-os-Montes e Alto Douro, 5000-801 Vila Real, Portugal; 8 Instituto de Telecomunicações, Escola Superior de Tecnologia e Gestão de Águeda, Universidade de Aveiro, Águeda, Portugal

**Keywords:** Pole vault, Blood lactate, Heart rate, Perceived exertion, Approach velocity, Step kinematics, Cardiology, Medical research, Physiology

## Abstract

Biomechanical analyses identify approach velocity—particularly in the final meters—as the primary mechanical determinant of vertical displacement in pole vault; yet how this velocity changes across repeated attempts performed below personal best height remains a practically important but largely untested question. As part of an applied field-based preparatory-phase protocol, we monitored the metabolic, cardiovascular, and perceptual responses of pole vaulters during four vault attempts performed 20 cm below their personal bests, and examined whether approach velocity was maintained in the final 5 m under these conditions. Eleven competitive pole vaulters (4 women, 7 men; age 20.3 ± 2.6 years) performed four consecutive vault attempts at heights 20 cm below their personal bests, with 3.5–4 min recovery intervals. Blood lactate (BL), heart rate (HR), and ratings of perceived exertion (RPE; Borg CR-10) were measured at predetermined time points throughout the protocol. Approach velocity and step kinematics (step length [SL], step frequency [SF], and contact time [CT]) over the final 5 m were derived from two-dimensional video analysis. Phase-related changes, load-velocity and step kinematic-velocity associations, subgroup interaction effects, and individual load change correlations were assessed using linear mixed-effects models (LMM) and Pearson correlation analyses. BL and HR showed no significant change between successive attempts (A_1_–A_4_; all p_Tukey_ > 0.05), whereas RPE increased significantly between A_3_ and A_4_. At A_4(11)_, neither BL nor RPE differed significantly from resting values (BL: *p*_*Tukey*_ = 0.375; RPE: : *p*_*Tukey*_ = 0.894); HR reached warm-up levels but remained significantly above rest (*p*_*Tukey*_ = 0.001). Approach velocity and all step parameters did not show a significant change throughout the four attempts (all *p* > .05). Neither exploratory model identified a significant relationship between velocity and physiological load indicators or step kinematics (all *p* > .05). Individual trajectory analysis revealed heterogeneity in velocity and kinematic strategies beneath the group-level mean. Subgroup interaction analyses revealed significant Attempt × Group effects for approach velocity in both the V_1_ (*F(3*,*27)* = 11.80, *p* < .001) and V_3_ (*F(3*,*24)* = 9.28, *p* < .001) models, with Velocity-Declining athletes demonstrating significantly greater velocity decrements at A_3_ and A_4_. Pole vaulters showed no detectable change in approach velocity across four consecutive attempts despite rising effort perception and progressive metabolic stress. The heterogeneity in individual kinematic strategies underscores the need for cautious interpretation of group-level findings.

## Introduction

**Fig. 1:**
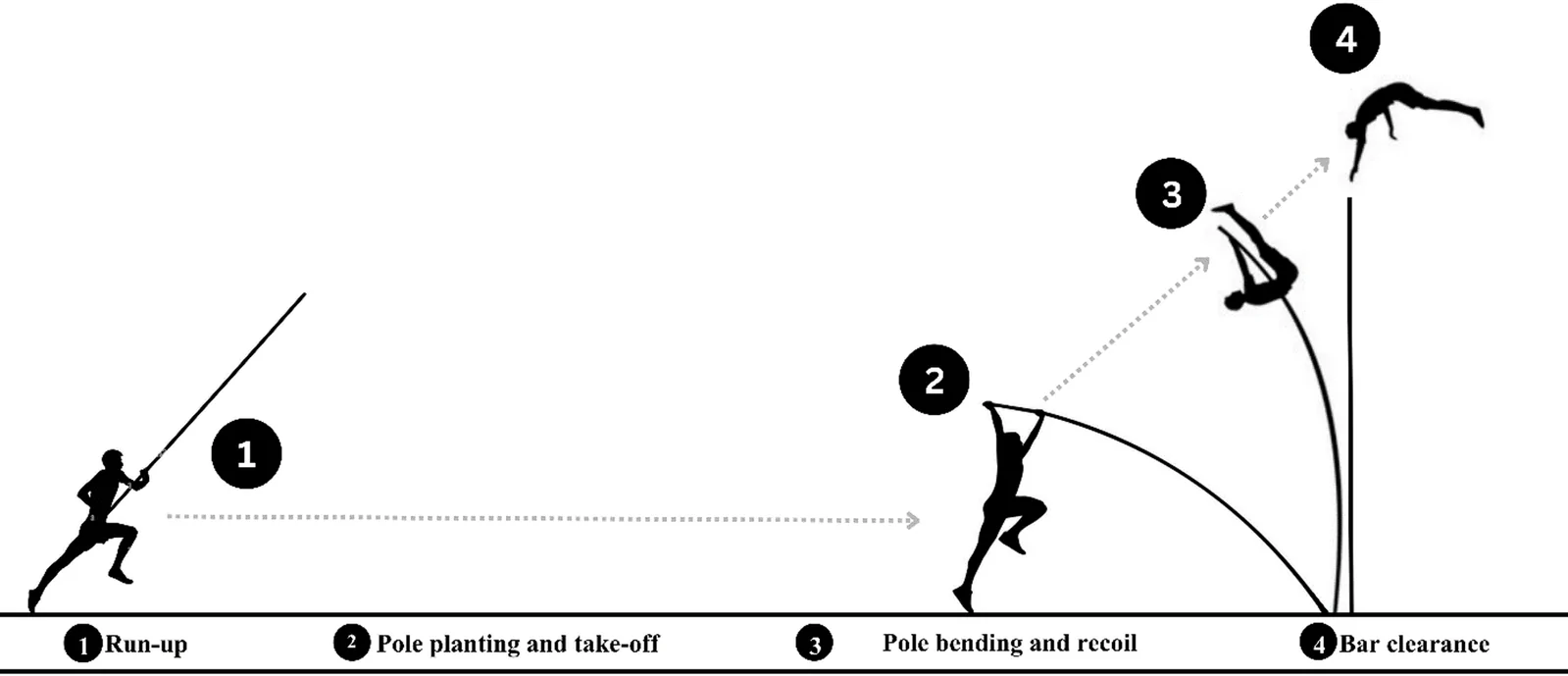
Pole vault phases.

Pole vault is a mechanically complex track and field event in which kinetic energy generated during the approach run is converted into potential energy through the pole-athlete interaction^1,2^. The vault comprises sequential phases: the approach run (run-up), pole plant and take-off, pole bending and recoil, and bar clearance^3^(Fig. 1). Approach-run velocity—particularly in the final meters—has been identified as a primary performance determinant, as greater horizontal velocity at take-off directly increases the height achievable during bar clearance^2,4^.

In competition, recovery intervals between attempts are dictated by the flow of the event rather than by athletes’ physiological needs, and no minimum interval is prescribed. The time allotted to initiate an attempt ranges from 1 to 5 min depending on the number of remaining competitors, and is fixed at 3 min for consecutive attempts by the same athlete^5^.

Research on repeated-sprint exercise has shown that mechanical performance may decline as physiological load accumulates^6,7^. Given that pole vault attempts are functionally analogous to repeated high-intensity intermittent efforts^8,9^, it is of practical interest how athletes sustain performance under the physiological strain imposed by short and variable recovery intervals. To the authors’ knowledge, however, no study has examined approach velocity and spatiotemporal step kinematics in conjunction with physiological and perceptual load in pole vault, nor characterised individual velocity response heterogeneity in relation to internal load accumulation.

Because the competitive structure of pole vault precludes standardization, we designed a controlled protocol with predetermined attempt number, recovery intervals, and bar height. Our aims were to characterize the temporal profiles of blood lactate^10^, heart rate^11^, and perceived exertion^12^ during this protocol, to examine short-term blood lactate and heart rate recovery kinetics following the final attempt, and to determine whether final 5-meter approach velocity and step kinematics were maintained across attempts. Additionally, we aimed to examine whether physiological and kinematic trajectories differed between athletes who maintained or improved approach velocity and those who exhibited velocity decline across attempts, and to develop a preliminary composite internal load monitoring index integrating the three physiological indicators. We hypothesized that both approach velocity and step kinematics would be maintained, and that physiological-load indicators would show no clear or strong association with velocity.

## Methods

### Participants

Eleven competitive pole vaulters (4 women, 7 men) volunteered to participate. Demographic and performance characteristics are presented in Table 1. All participants were registered with the Turkish Athletics Federation and had been competing for at least three years; six held national team status and two were Turkish record holders. Accordingly, the findings reflect a high-performance sample rather than the general athletic population. Given the small and unequal sex distribution, sex was not included as a covariate in any analysis; this is acknowledged as a limitation.

Ethical approval was granted by the Marmara University Clinical Research Ethics Committee (Protocol No: 09.2021.800; approved June 4, 2021). The study was conducted in accordance with the Declaration of Helsinki, and written informed consent was obtained from all participants prior to enrolment.

Data collection was conducted during the general preparation phase of each participant’s annual training cycle—a period when athletes are well-trained but not yet at peak competitive readiness—to minimise the potential influence of acute fatigue on measurements^13^.

As no direct LMM-equivalent power procedure exists, an a priori power analysis was performed using a repeated-measures ANOVA approximation (1 group, 9 measurements) in G*Power 3.1^14^, yielding a required sample size of 11 (f = 0.30, α = 0.05, 80% power). This analysis was designed to detect a medium omnibus effect and was not tailored to the velocity outcome or the exploratory covariance models.

The sample size is consistent with the limited availability of high-level pole vaulters and aligns with previous work in this discipline^3,15^. Nevertheless, statistical power for individual contrasts—particularly for approach velocity—remains limited at *n* = 11, and non-significant velocity findings should be interpreted with appropriate caution.

### Study design

The protocol was modelled on the structure of pole vault competition, including athletes’ pre-competition warm-up routines. Because numerous parameters are uncontrollable in a real competition setting, bar height, number of attempts, and recovery intervals were held constant to standardise repeated measurements across participants. The findings therefore reflect a controlled preparatory-phase workload under conditions designed to maximise cumulative load accumulation rather than replicate maximal competitive stress.

### Experimental protocol

**Fig. 2:**
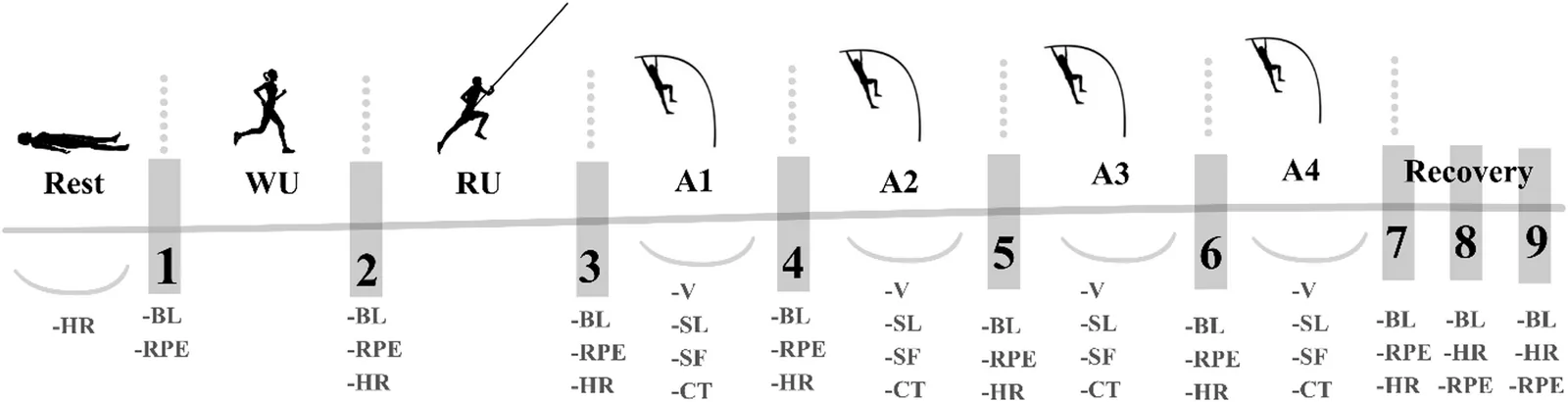
Experimental protocol.

The protocol comprised four consecutive stages (Fig. 2):

- Rest: Participants rested supine for 5 min to establish baseline physiological conditions.
- Warm-up (WU): Athletes completed a standardised warm-up consisting of 10 min of low-intensity aerobic running followed by pole vault-specific drills (e.g., short approach runs, pole-carry drills, and planting exercises). Although running time was similar across participants, total warm-up duration varied according to individual preparation needs (mean: 40.9 ± 13.4 min). No significant correlation was observed between warm-up duration and final 5-m approach velocity across attempts (all Spearman’s ρ < 0.609, all *p* > .05); warm-up duration was therefore not included as a model covariate. Participants then rested passively for 4 min.
- Run-Up (RU): Participants completed a 30-meter approach run without take-off or bar clearance, followed by a 4–6 min recovery interval.
- Attempts (A _1_–A _4_ ): Four consecutive vault attempts were performed. Bar heights were set at 20 cm below each athlete’s personal best, a margin selected to eliminate psychological performance anxiety and ensure technical execution consistency rather than to reduce physiological demand. In elite pole vault, approach velocity, pole carry mechanics, and take-off execution are performed with maximal technical intent regardless of bar height^3^. The 20 cm below personal best margin additionally ensured successful bar clearance on every attempt, eliminating technical failure as a source of kinematic and physiological variability. All recorded attempts resulted in successful clearance; no attempt was excluded from analysis on technical grounds. Recovery intervals were fixed at 3.5–4 min, representing the minimum competitive recovery window observed in elite pole vault competition. In real competition, inter-attempt intervals vary considerably—from under five minutes to over 30 min—depending on the number of competitors, the total duration of the competition session, and the inherent dynamics of the progressive bar-raising format^5^. This fixed interval was therefore deliberately selected to maximise cumulative internal load accumulation across attempts.

This protocol was designed to examine performance output under controlled preparatory-phase conditions and was not intended to replicate maximal competitive stress; this distinction is addressed further in the Limitations section.

### Notation

To ensure chronological clarity and typographic consistency, time points are denoted using phase-based labels followed by the measurement minute in parentheses. For example, 3 min post-warm-up is written as WU_3_. Vault attempts are labelled A_1_ to A_4_; where applicable, the post-effort measurement minute appears in parentheses. For instance, the 3-min post-effort measurement following attempt 1 is A_1(3)_, and the 7th and 11th minute recovery points of the final attempt are A_4(7)_ and A_4(11)_.

### Blood lactate assessment

Capillary blood samples were obtained from the earlobe and analysed using a portable lactate analyser with established field validity and reliability (Lactate Scout; EKF Diagnostics, Germany)^16^. Nine samples were collected per participant: at baseline (after 5 min of supine rest), 3 min after warm-up, 3 min after the 30-m approach run, 3 min after each of the first three vault attempts, and at 3, 7, and 11 min after the fourth attempt.

The 3-min post-effort sampling point was selected because blood lactate concentration typically peaks approximately 3 min after the cessation of short-term maximal exercise^10^. It should be noted that the approach run and vault attempts differ in their biomechanical and metabolic demands: the approach run involves pole carriage only, whereas vault attempts additionally encompass take-off, pole bending and recoil, and bar clearance. This distinction should be considered when interpreting the findings.

### Heart rate assessment

RR intervals were recorded continuously throughout the protocol using a chest-strap monitor (Polar H10; Polar Electro, Finland), and mean HR was derived from these intervals using Kubios HRV Scientific software (Kubios Oy, Finland). A 5-minute baseline window was analysed at rest. For all subsequent efforts, mean HR was extracted at the 3rd minute post-effort to align temporally with blood lactate sampling. For the final attempt, additional windows at 7 and 11 min were included to characterise recovery kinetics.

Given the brief duration of individual vault attempts (~ 5–6 s), HR was assessed post-effort rather than during the attempt itself, for two methodological reasons. First, the last 5 m run-up phase lasted approximately 1 s per attempt, during which only 0–1 cardiac cycles occurred; extracting a meaningful HR estimate from 0 to 1 RR intervals is neither statistically valid nor physiologically interpretable as an index of cardiovascular load. Second, cardiac response operates with an inherent temporal delay of approximately 10–20 s relative to the mechanical stimulus; heart rate during the final 5 m therefore reflects carry-over from the preceding effort phase rather than the instantaneous cardiovascular demand of the run-up. Post-effort HR values therefore reflect cumulative cardiovascular activation rather than instantaneous effort cost, and are temporally aligned with blood lactate sampling at the 3-minute post-effort^11^.

### Rating of perceived exertion (RPE)

Prior to testing, participants were familiarised with the Borg Category Ratio scale (CR-10^12^) and instructed to rate their overall perceived effort at each measurement point. RPE was recorded immediately after each phase and attempt, and at 7 and 11 min following the final attempt.

### Approach-run velocity and step kinematics

Each vault attempt was recorded at 120 frames s^−1^ using a GoPro Hero 4 camera (GoPro Inc., San Mateo, CA, USA), mounted on a tripod at 1.5 m height and positioned perpendicular to the runway. Although photocell-based systems offer greater temporal precision, two-dimensional (2D) video analysis was preferred as an ecologically valid approach^17^. Final 5-m approach-run velocity was calculated using Tracker^18^, with the midpoint of the athlete’s hips digitised frame-by-frame as a proxy for whole-body centre of mass^17^.

To assess measurement precision, 20% of trials (*n* = 9) were re-digitised by the same analyst after a 1-day interval. Intra-rater reliability was excellent (ICC 0.982, 95% CI 0.921–0.996), with a typical error of 0.09 m s^−1^ (~ 1.9% of mean velocity).

Step kinematics over the final 5 m were derived from foot contact points identified in the same recordings. Step length (SL) was defined as the horizontal distance between two consecutive initial contact points; step frequency (SF) as the inverse of the time between two consecutive initial contacts; and contact time (CT) as the time from initial contact to toe-off^19^. Where two full steps were visible in the final 5 m, the average of those two steps was reported; where three were visible, the average of three was used. A single SL, SF, and CT value was thus obtained for each athlete per attempt.

### Statistical analysis

All statistical analyses were conducted using Jamovi software (Version 2.3.6). Descriptive statistics are presented as mean ± SD, minimum and maximum values where appropriate. Statistical significance was set at α = 0.05.

Given the substantial inter-individual variability inherent in repeated-measures pole vault data, linear mixed-effects models (LMM) were used throughout^14^. In all models, Phase was entered as the fixed effect and Athlete as the random intercept. Random slope inclusion did not improve model fit in any case (likelihood ratio tests: all *p* > .10) and was therefore omitted.

Model assumptions were evaluated visually using Q–Q plots, given the small sample size^14^; no meaningful deviation from normality or homoscedasticity was observed. For the velocity model, formal tests were also applied: neither the Kolmogorov–Smirnov test (D = 0.087, *p* = .865) nor the Shapiro–Wilk test (W = 0.975, *p* = .432) indicated a violation of normality.

Fixed effects were estimated using the Kenward–Roger degrees of freedom approach, and post-hoc pairwise comparisons were Tukey-corrected based on estimated marginal means. Model fit was indexed by marginal R^2^ (R^2^m; variance explained by fixed effects) and conditional R^2^ (R^2^c; variance explained by the full model)^20^. As LMMs do not yield conventional standardised effect sizes, and given the small sample and exploratory nature of several comparisons, effect magnitude is characterised by R^2^ values and 95% confidence intervals throughout.

As a supplementary precision check, observed attempt-to-attempt velocity differences (range: 0.01–0.13 m s^−1^) were compared against the typical error of the video analysis procedure (0.09 m s^−1^). Most observed differences fall at or below this measurement noise threshold, providing additional support for the interpretation that no meaningful change in approach velocity occurred across attempts.

Two exploratory LMMs were constructed to examine load-velocity and kinematic-velocity associations. In the first (V_2_), HR and BL were entered as continuous covariates alongside Attempt to examine their association with approach velocity. In the second (V_3_), SL, SF, and CT were entered as continuous covariates alongside Attempt for the same purpose.

To characterise individual trajectories, ordinary least-squares regression slopes were calculated for V1, SL, SF, and CT across A_1_–A_4_ for each athlete, with attempt number as the independent variable (A_1_ = 1, A_2_ = 2, A_3_ = 3, A_4_ = 4). Athletes were classified using thresholds set at one-third of the typical error for each parameter, following Atkinson and Nevill^21^: ± 0.030 m/s/attempt for V1, ± 0.007 m/attempt for SL, ± 0.033 steps/s/attempt for SF, and ± 0.002 s/attempt for CT. Slopes exceeding the positive threshold were classified as improver (↑), those exceeding the negative threshold as decliner (↓), and those within the threshold as maintainer (↔). This classification is strictly descriptive and exploratory, and is not intended for statistical inference.

For inferential subgroup analyses, the original three-category V_1_ classification (Improver/Maintainer/Decliner) was collapsed into a binary grouping to preserve statistical power given the limited sample size: athletes with a V_1_ slope ≥ 0 were classified as Velocity-Stable (combining Improvers and Maintainers; *n* = 7) and those with a V_1_ slope < 0 as Velocity-Declining (*n* = 4). A zero slope constitutes a theoretically meaningful threshold distinguishing athletes who maintained or improved approach velocity from those who exhibited measurable decline. This binary classification was applied consistently across all kinematic and physiological parameters to ensure analytical coherence.

To formally examine whether physiological and kinematic trajectories differed between velocity subgroups, Phase × Group and Attempt × Group interaction terms were added to the primary linear mixed models fitted to the full sample (*n* = 11), with Group entered as a fixed effect and Athlete_ID retained as a random intercept. Additionally, Pearson product-moment correlation coefficients were computed to examine associations between individual V_1_ slope and the magnitude of change in BL (ΔBL = BL_A4_ − BL_A1_), HR (ΔHR = HR_A4_ − HR_A1_), and RPE (ΔRPE = RPE_A4_ − RPE_A1_).

As a supplementary descriptive analysis, a Composite Load Index (CLI) was developed to integrate the three internal load indicators into a single recovery-tracking metric. Each indicator was normalised to the individual peak value observed within the protocol (peak HR, peak BL, and peak RPE), and weighted according to empirically observed differential recovery rates across the post-A_4_ window (A_4(3)_ to A_4(11)_): HR demonstrated the slowest recovery rate and received the highest weight (w = 0.42), followed by BL (w = 0.30) and RPE (w = 0.28). The CLI formula was defined as:

$${\rm{CLI }} = 0.{\rm{42 }} \times {\rm{ }}\left( {{\rm{H}}{{\rm{R}}_{{\rm{observed}}}}/{\rm{ H}}{{\rm{R}}_{{\rm{peak}}}}} \right){\rm{ }} + {\rm{ }}0.{\rm{3}}0{\rm{ }} \times {\rm{ }}\left( {{\rm{B}}{{\rm{L}}_{{\rm{observed}}}}/{\rm{ B}}{{\rm{L}}_{{\rm{peak}}}}} \right){\rm{ }} + {\rm{ }}0.{\rm{28 }} \times {\rm{ }}\left( {{\rm{RP}}{{\rm{E}}_{{\rm{observed}}}}/{\rm{ RP}}{{\rm{E}}_{{\rm{peak}}}}} \right)$$

A CLI value of 1.00 represents each athlete’s peak load point within the protocol; decreasing values indicate progressive recovery. The CLI is presented as a preliminary exploratory framework and has not been prospectively validated.

## Results

Demographic and performance characteristics of the participants are presented in Table 1.

**Table 1:** Descriptive statistics of the athletes’ characteristics.

Variable	Mean ± SD	Min	Max
Height (m)	1.79 ± 0.08	1.68	1.93
Weight (kg)	66.3 ± 6.38	54.0	73.0
Age (years)	20.3 ± 2.57	18	24
Sport age (years)	7.64 ± 3.04	3	13
PB (m)	4.12 ± 0.77	3.00	5.21
Approach (m)	18.0 ± 3.41	14.0	24.4

SD, Standard deviation; Min, Minimum; Max, Maximum; PB, Personal best; Approach, Approach-run distance.

**Table 2:** Model fit indices and fixed effects omnibus tests for post-effort physiological markers and kinematic parameters.

Variable	*R*²m	*R*²c	F(df1, df2)	*p*
Physiological parameters				
BL-Phase	0.196	0.705	*F(8*,* 80) =* 6.95	< 0.001
HR-Phase	0.189	0.796	*F(8*,* 80) =* 11.6	< 0.001
RPE-Phase	0.372	0.372	*F(8*,* 80) =* 7.00	< 0.001
Exploratory mode-1				
V_2_-Attempt	0.222	0.222	*F(3*,* 28) =* 1.38	0.270
V_2_-BL	–	–	*F(1*,* 28) =* 1.27	0.268
V_2_-HR	–	–	*F(1*,* 28) =* 3.20	0.084
Velocity-kinematic parameters				
V_1_-Attempt	0.004	0.908	*F(3*,* 30) =* 1.33	0.334
SL-Attempt	0.006	0.948	*F(3*,* 30) =* 1.32	0.232
SF-Attempt	0.004	0.860	*F(3*,* 30) =* 0.48	0.699
CT-Attempt	0.009	0.812	*F(3*,* 30) =* 0.94	0.432
Exploratory model-2				
V_3_-Attempt	0.074	0.923	*F(3*,* 27) =* 1.32	0.288
V_3_-SL	–	–	*F(1*,* 27) =* 0.72	0.403
V_3_-CT	–	–	*F(1*,* 27) =* 0.35	0.554
V_3_-SF	–	–	*F(1*,* 27) =* 0.80	0.379

R^2^m , Marginal R^2^; *R*^2^c, Conditional R^2^; BL, Blood lactate; HR, Heart rate; RPE, Ratings of perceived exertion; SL, Step length; SF, Step frequency; CT, Contact time; V_1_, Last 5-meter velocity; V_2_, Exploratory model 1 (physiological predictors); V_3_, Exploratory model 2 (kinematic predictors). For the exploratory velocity models;* R*^2^m and R^2^c values apply to the full model and are reported once; dashes indicate that separate R^2^ values are not computed for individual covariate fixed effects. Phase fixed effect was used for physiological markes, while Attempt fixed effect was used for velocity and kinematics.

**Fig. 3:**
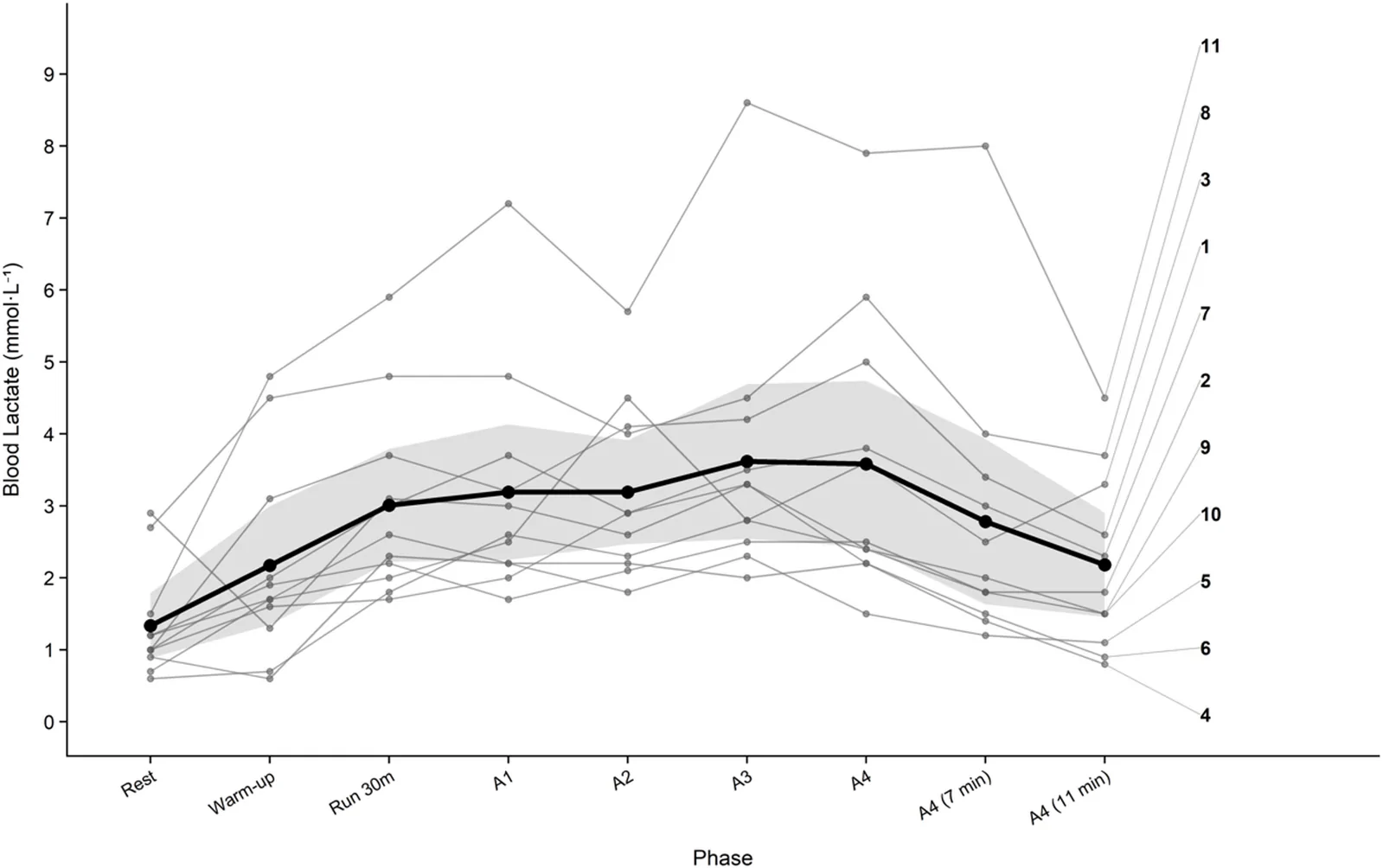
Post-effort capillary blood lactate kinetics across experimental and recovery phases (Note. Individual participant trajectories are indexed and labeled by Athlete ID. The thick solid black line represents the estimated marginal population mean, with the shaded region indicating the corresponding 95% confidence intervals.)

Phase had a significant effect on BL (Table 2; *F(8*,*80)* = 6.95, *p* < .001; *R²m* = 0.196, *R²c* = 0.705). Estimated marginal means (EMMs) and pairwise comparisons are presented in Table 3; individual and group-level trajectories are shown in Fig. 3. No significant difference was observed between consecutive vault attempts (all *p*_*Tukey*_ > 0.05; Table 3). At A_4(7)_, BL did not differ significantly from WU_3_ (*p*_*Tukey*_ = 0.781), and by A_4(11)_ it had returned to values comparable to rest (*p*_*Tukey*_ = 0.375; Table 3). A significant difference was nonetheless observed between A_4(3)_ and A_4(11)_ values (*p*_*Tukey*_ = 0.002; Table 3).

**Table 3:** Estimated marginal means and pairwise comparisons for physiological markers across experimental phases.

Phase	BL		HR		RPE	
	Mean (95% CI)		Mean (95% CI)		Mean (95% CI)	
R	1.34 (0.365–2.31)		86.7 (73.8–99.5)		2.64 (1.71–3.56)	
WU_3_	2.17 (1.201–3.14)		113.9 (101.1–126.7)		2.82 (1.89–3.74)	
RU_3_	3.01 (2.038–3.98)		112.7 (99.9–125.6)		3.82 (2.89–4.74)	
A_1(3)_	3.19 (2.219–4.16)		114.1 (101.2–126.9)		4.27 (3.35–5.20)	
A_2(3)_	3.19 (2.219–4.16)		115.1 (102.3–127.9)		5.00 (4.07–5.93)	
A_3(3)_	3.62 (2.647–4.59)		113.7 (100.9–126.5)		4.82 (3.89–5.74)	
A_4(3)_	3.58 (2.610–4.55)		119.8 (107.0–132.6)		6.09 (5.16–7.02)	
A_4(7)_	2.78 (1.810–3.75)		111.9 (99.0–124.7)		4.82 (3.89–5.74)	
A_4(11)_	2.18 (1.210–3.15)		104.3 (91.5–117.2)		3.45 (2.53–4.38)	
Comparison	diff	*p*_***Tukey***_	diff	*p*_***Tukey***_	diff	*p*_***Tukey***_
R – WU_3_	− 0.83	0.071	− 27.255	< 0.001	− 0.182	1.000
R_3_ – A_4(3)_	− 2.24	< 0.001	-33.157	< 0.001	− 3.455	< 0.001
R_3_ – A_4(7)_	− 1.44	0.006	− 25.186	< 0.001	− 2.182	0.009
R_3_ – A_4(11)_	− 0.84	0.375	− 17.661	0.001	− 0.818	0.894
WU_3_ – RU_3_	− 0.83	0.071	1.174	1.000	− 1.000	0.183
WU_3_ – A_4(3)_	− 1.40	0.008	− 5.902	0.877	− 3.273	< 0.001
WU_3_ – A_4(7)_	− 0.60	0.781	2.069	1.000	-2.000	0.022
WU_3_ – A_4(11)_	− 0.009	1.000	9.594	0.327	− 0.636	0.973
RU_3_ – A_1(3)_	− 0.18	0.999	− 1.316	1.000	− 0.455	0.953
A_1(3)_ – A_2(3)_	0.00	1.000	− 1.058	1.000	− 0.727	0.597
A_1(3)_ – A_3(3)_	− 0.42	0.932	0.340	1.000	− 0.545	0.965
A_1(3)_ – A_4(3)_	− 0.39	0.972	− 5.761	0.890	− 1.818	0.023
A_2(3)_ – A_3(3)_	− 0.42	0.823	1.398	1.000	0.182	1.000
A_2(3)_ – A_4(3)_	− 0.39	0.958	− 4.703	0.962	− 1.091	0.358
A_3(3)_ – A_4(3)_	0.03	1.000	− 6.101	0.793	− 1.273	0.030
A_4(3)_ – A_4(7)_	0.80	0.099	7.971	0.472	1.273	0.030
A_4(3)_ – A_4(11)_	1.40	0.002	15.496	0.007	2.636	< 0.001
A_4(7)_ – A_4(11)_	0.60	0.422	7.525	0.552	1.364	0.015

BL, Blood lactate, HR, Heart rate, RPE, Ratings of perceived exertion; R, Rest; WU, Warm-up; RU, Run-up, A_1_–A_4_; Attempt 1, Attempt 4; Parenthetical numbers _(3), (7),_ and _(11)_ denote the specific measurement minute during the post-effort phase. Acute RPE was obtained immediately post-effort (0 min) to capture instantaneous perceptual effort, whereas BL and HR were sampled at the 3rd minute to ensure physiological stabilization and metabolic peak capture. For all pairwise comparisons, degrees of freedom were estimated using Kenward-Roger approximation df = 80.0).

**Fig. 4:**
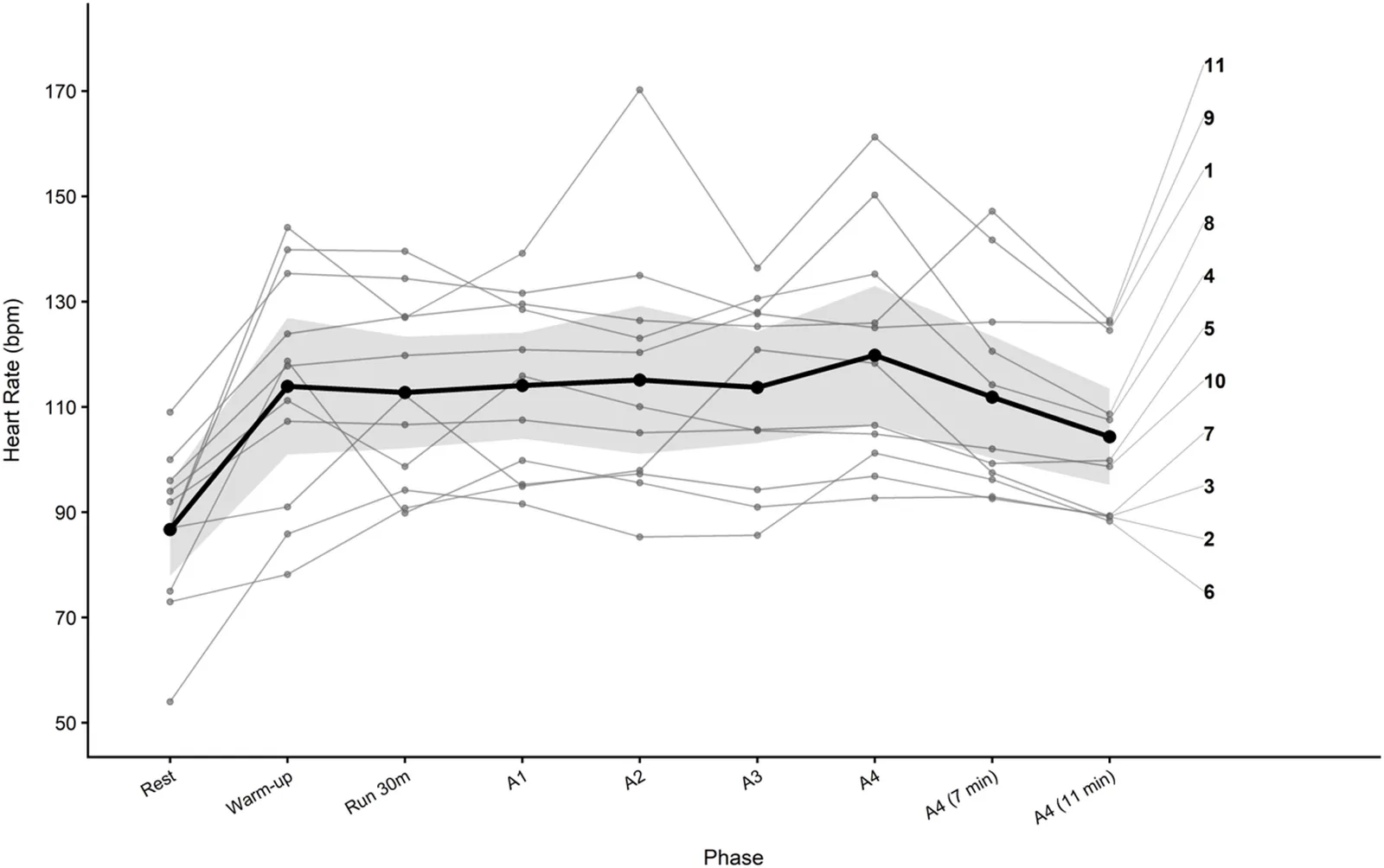
Post-effort heart rate kinetics across experimental and recovery phases (Note. Individual participant trajectories are indexed and labeled by Athlete ID. The thick solid black line represents the estimated marginal population mean, with the shaded region indicating the corresponding 95% confidence intervals).

Phase had a significant effect on HR (Table 2; *F(8*,*80)* = 11.6, *p* < .001; *R²m* = 0.189, *R²c* = 0.796). EMMs and pairwise comparisons are presented in Table 3, and individual and group-level variation is shown in Fig. 4. No significant differences were observed between consecutive attempts (*all p*_*Tukey*_ > 0.05; Table 3). At A_4(11)_, HR was comparable to warm-up levels (*p*_*Tukey*_ = 0.327) yet remained significantly above resting values (*p*_*Tukey*_ = 0.001).

**Fig. 5:**
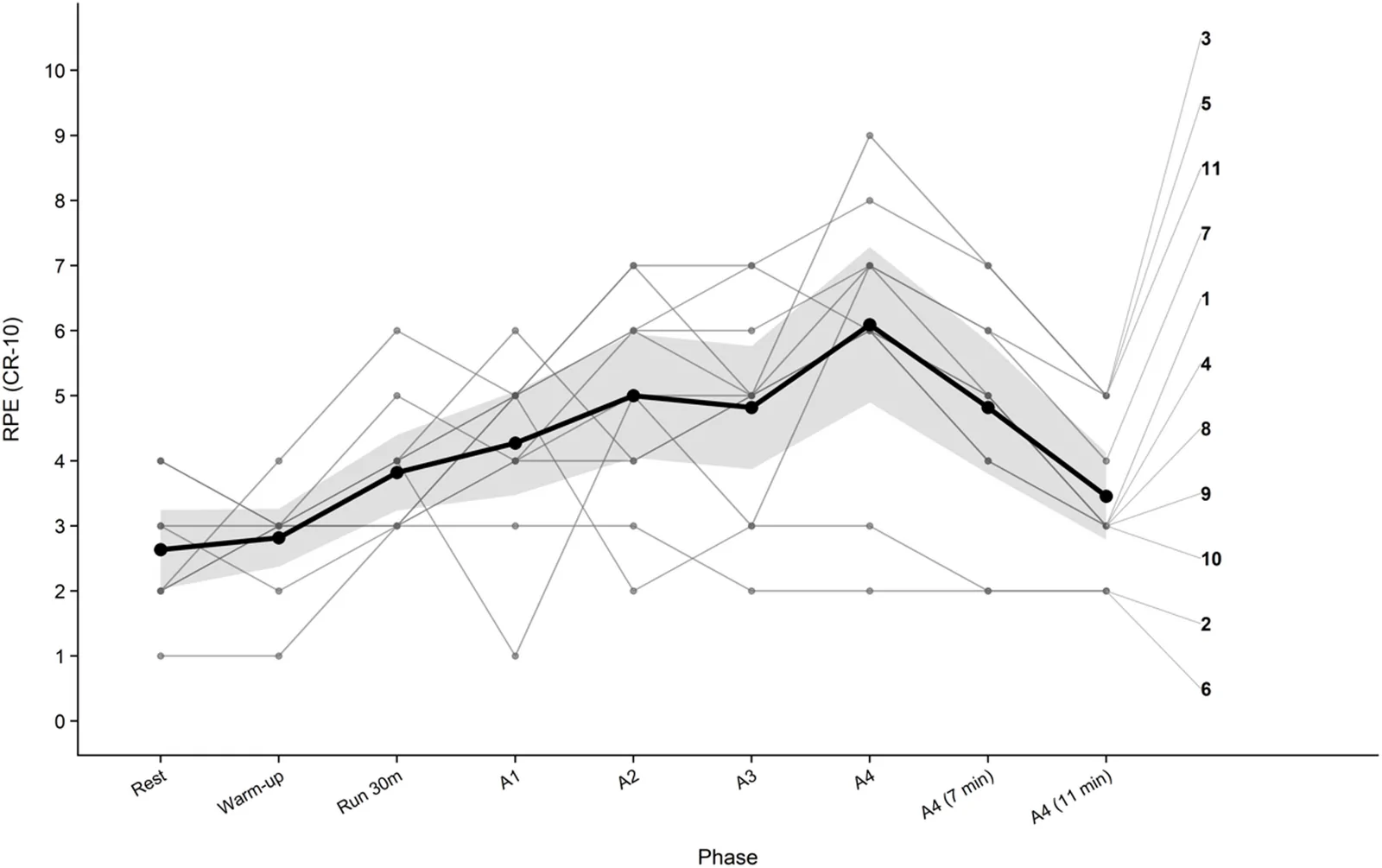
Perceptual rating of perceived exertion (CR-10) Kinetics across experimental and recovery phases (Note. Individual participant trajectories are indexed and labeled by Athlete ID. The thick solid black line represents the estimated marginal population mean, with the shaded region indicating the corresponding 95% confidence intervals).

RPE was recorded immediately post-effort (0 min) and at 7 and 11 min following the final attempt. Phase had a significant effect on RPE (Table 2; *F(8*,*80) =* 7.00, *p* < 0.001; *R²m* = 0.372, *R²c* = 0.372). EMMs and pairwise comparisons are presented in Table 3; individual and group-level trajectories are shown in Fig. 5. RPE increased progressively across attempts, with only the A_3(3)_ – A_4(3)_ comparison reaching significance among consecutive attempts (*p*_*Tukey*_ = 0.030; Table 3). Following the final attempt, RPE declined stepwise: significant decreases were observed between A_4(3)_ and A_4(7)_ (*p*_*Tukey*_ = 0.030; Table 3) and between A_4(7)_ and A_4(11)_ (*p*_*Tukey*_ = 0.015). By A_4(11)_, RPE did not differ significantly from either rest (*p*_*Tukey*_ = 0.894) or WU_3_ (*p*_*Tukey*_ = 0.973).

**Table 4:** Estimated marginal means and pairwise comparisons for velocity and step kinematics across repeated attempts.

Attempt	V_1_		SL		SF		CT	
	Mean (95% CI)		Mean (95% CI)		Mean (95% CI)		Mean (95% CI)	
A_1_	6.67 (6.11–7.23)		1.27 (1.14–1.40)		4.32 (3.94–4.70)		0.145 (0.131–0.158)	
A_2_	6.68 (6.12–7.24)		1.30 (1.16–1.43)		4.23 (3.85–4.61)		0.149 (0.135–0.163)	
A_3_	6.71 (6.15–7.27)		1.30 (1.16–1.43)		4.25 (3.87–4.63)		0.149 (0.136–0.163)	
A_4_	6.57 (6.01–7.13)		1.31 (1.18–1.45)		4.24 (3.86–4.62)		0.149 (0.135–0.162)	
Comparison	diff	p_**Tukey**_	diff	p_**Tukey**_	diff	p_**Tukey**_	diff	p_**Tukey**_
A_1_ – A_2_	− 0.01	0.999	− 0.027	0.495	0.087	0.678	− 0.00	0.407
A_1_ – A_3_	− 0.04	0.964	− 0.027	0.498	0.075	0.823	0.00	0.514
A_1_ – A_4_	0.09	0.774	− 0.040	0.189	0.078	0.814	0.00	0.678
A_2_ – A_3_	− 0.03	0.968	− 0.000	1.000	− 0.011	0.999	− 0.00	1.000
A_2_ – A_4_	0.10	0.661	− 0.013	0.905	− 0.008	1.000	0.00	0.999
A_3_ – A_4_	0.13	0.259	− 0.012	0.910	0.003	1.000	0.00	0.995

V_1_, Last 5-m approach velocity; SL, Step length; SF, Step frequency; CT, Contact time; A_1_–A_4_, Attempt 1 to Attempt 4; diff, mean difference; CI, Confidence interval. For all pairwise comparisons, degrees of freedom were estimated using Kenward–Roger approximation df = 30.0).

**Fig. 6:**
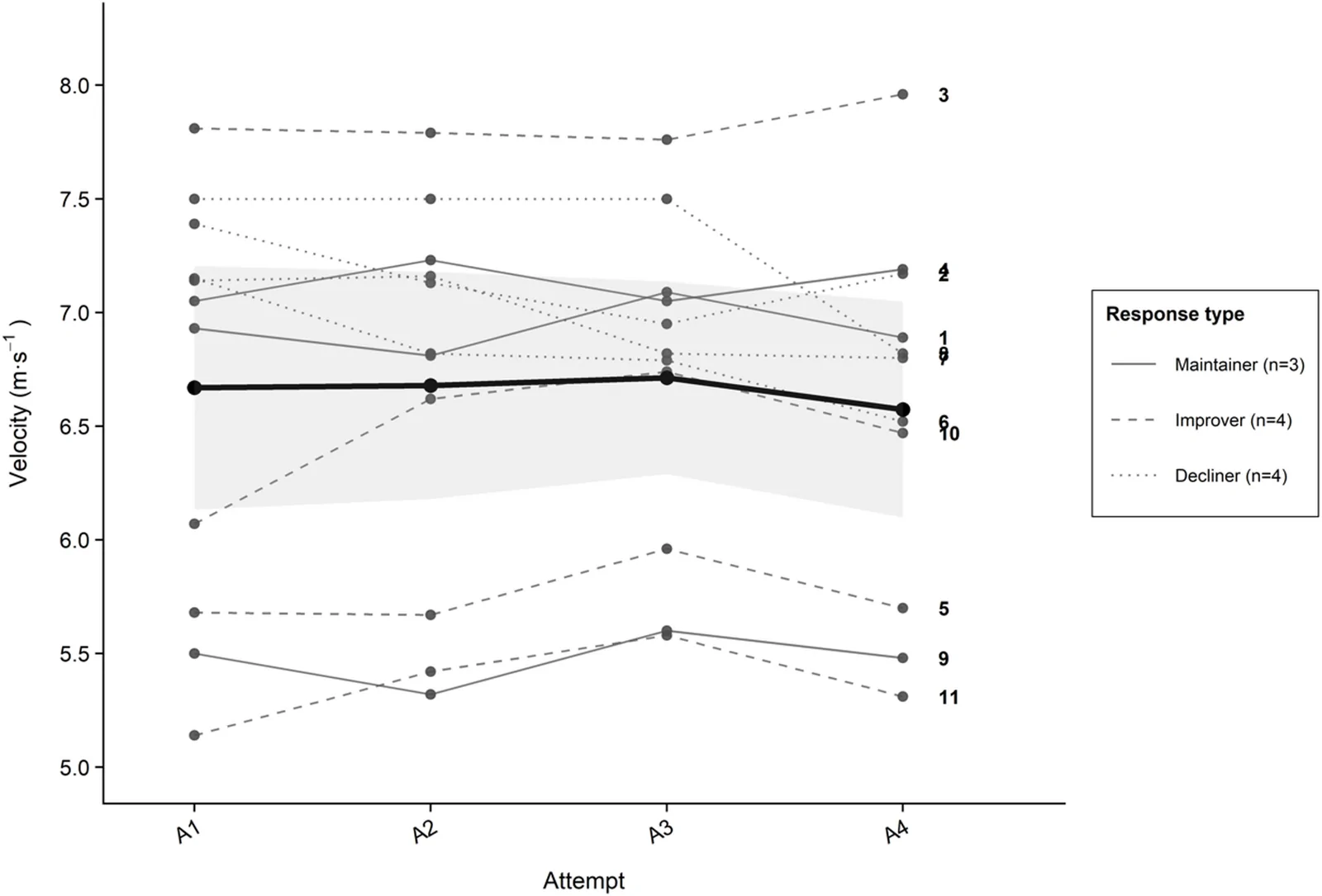
Last 5-meter approach velocity (V_1_) trajectories and slope-based responder classifications across attempts (Note. Individual classifications reflect parameter-specific slope thresholds and are independent of the V_1_-based velocity subgroup classification. Individual trajectories are indexed by Athlete ID. Line styles partition participants based on their slope-derived analytical classification: solid gray lines for Maintainers (*n* = 3), dashed lines for Improvers (*n* = 4), and dotted lines for Decliners (*n* = 4). The thick solid black line tracks the overall population average flanked by its 95% confidence interval band).

**Fig. 7:**
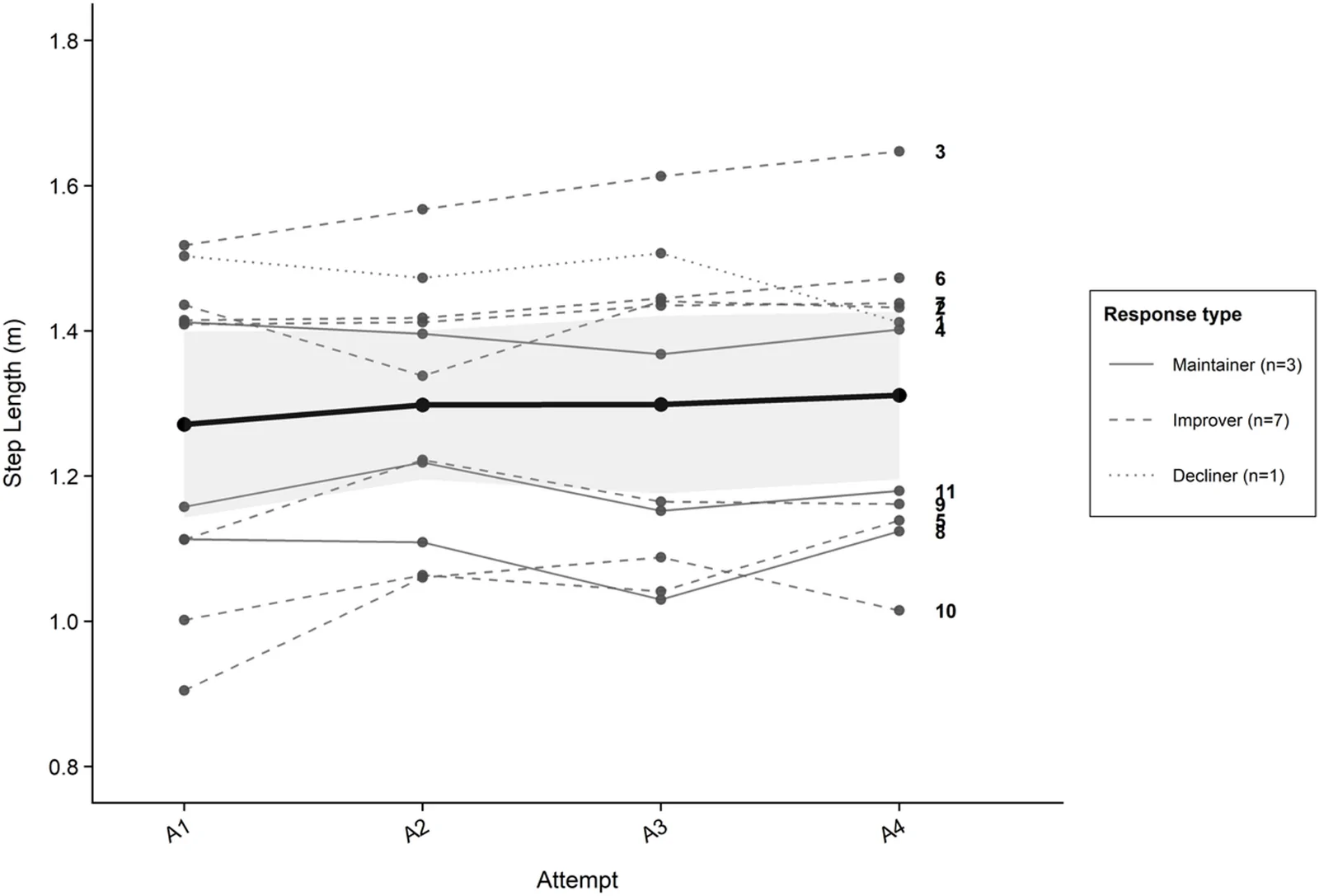
Spatiotemporal step length (SL) trajectories and slope-based classifications across attempts (Note. Individual classifications reflect parameter-specific slope thresholds and are independent of the V_1_-based velocity subgroup classification. Individual trajectories are indexed by Athlete ID. Line styles partition participants based on their slope-derived analytical classification: solid gray lines for Maintainers (*n* = 3), dashed lines for Improvers (*n* = 7), and dotted lines for Decliners (*n* = 1). The thick solid black line tracks the overall population average flanked by its 95% confidence interval band).

**Fig. 8:**
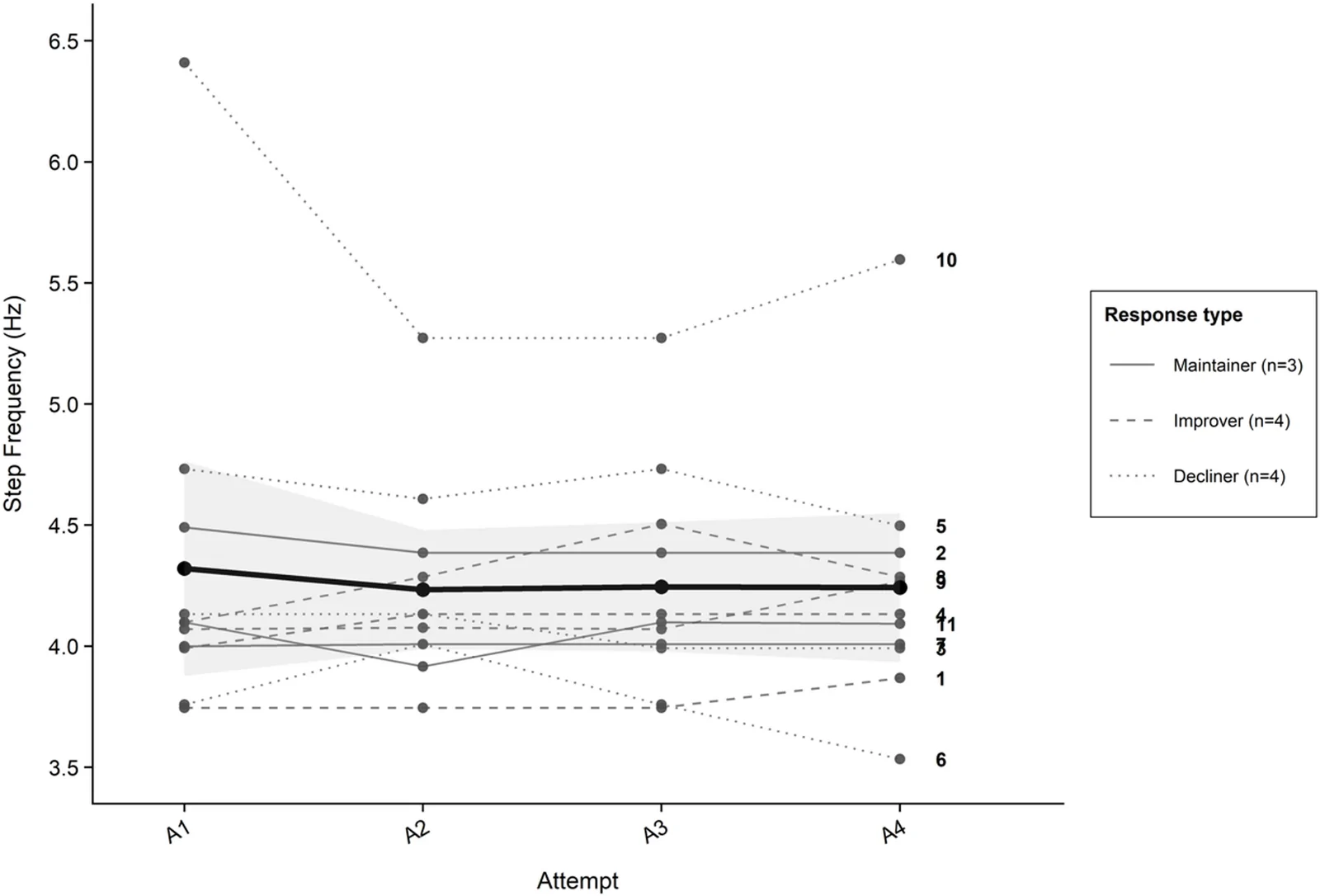
Spatiotemporal step frequency (SF) trajectories and slope-based classifications across attempts (Note. Individual classifications reflect parameter-specific slope thresholds and are independent of the V_1_-based velocity subgroup classification. Individual trajectories are indexed by Athlete ID. Line styles partition participants based on their slope-derived analytical classification: solid gray lines for Maintainers (*n* = 3), dashed lines for Improvers (*n* = 4), and dotted lines for Decliners (*n* = 4). The thick solid black line tracks the overall population average flanked by its 95% confidence interval band).

**Fig. 9:**
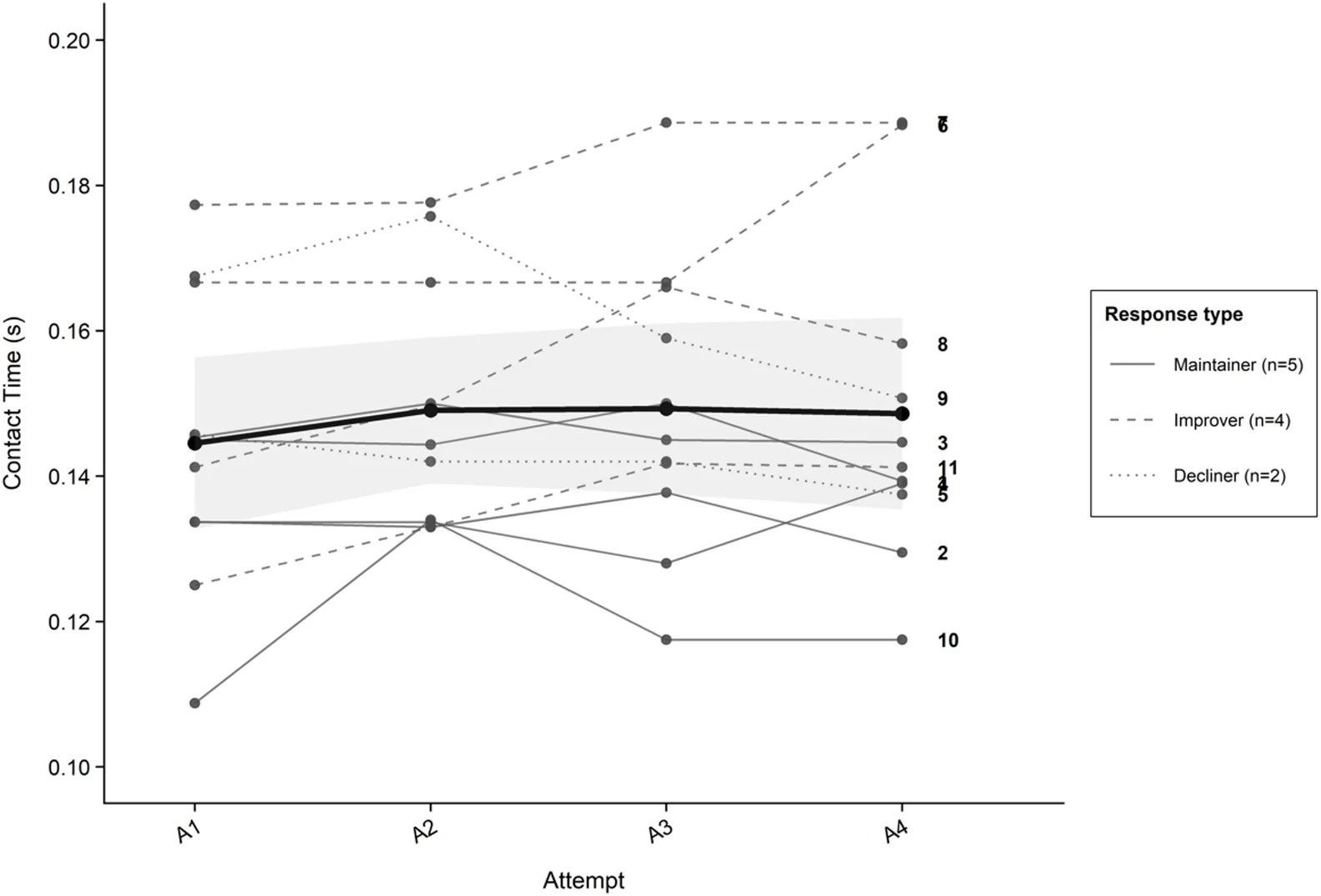
Ground contact time (CT) trajectories and slope-based classifications across attempts (Note. Individual classifications reflect parameter-specific slope thresholds and are independent of the V_1_-based velocity subgroup classification. Individual trajectories are indexed by Athlete ID. Line styles partition participants based on their slope-derived analytical classification: solid gray lines for Maintainers (*n* = 5), dashed lines for Improvers (*n* = 4), and dotted lines for Decliners (*n* = 2). The thick solid black line tracks the overall population average flanked by its 95% confidence interval band).

Phase had no significant effect on V_1_ (*F(3*,*30)* = 1.33, *p* = .334; *R²m* = 0.004, *R²c* = 0.908) or any step parameter (**SL;** *F(3*,*30)* = 1.32, *p* = .232; *R²m* = 0.006, *R²c* = 0.948, **SF;** *F(3*,*30)* = 0.48, *p* = .699; *R²m* = 0.004, *R²c* = 0.860, **CT**; *F(3*,*30)* = 0.94, *p* = .432; *R²m* = 0.009, *R²c* = 0.812) (Table 2). EMMs and pairwise comparisons are presented in Table 4; individual and group-level trajectories are shown in Figs. 6, 7, 8 and 9.

The substantial discrepancy between marginal and conditional R^2^ values across the V_1_, SL, SF, and CT models indicates that inter-individual differences, rather than phase effects, accounted for the vast majority of explained variance. Individual slope-based classifications are presented in Table 5; Figs. 6, 7, 8 and 9. For V_1_, 3 athletes were classified as Maintainer (27.3%), 4 as Decliner (36.4%), and 4 as Improver (36.4%). For SL, 3 were Maintainers (27.3%), 1 Decliner (9.1%; Athlete 1), and 7 Improvers (63.6%). SF showed an equal split between Decliners and Improvers (each 36.4%), with 3 Maintainers (27.3%). For CT, 5 athletes were classified as Maintainer (45.5%), 2 as Decliner (18.2%), and 4 as Improver (36.4%).

**Table 5:** Individual participant trajectories, linear regression slopes, and responder classifications for last 5-m velocity and step kinematics across repeated attempts.

ID	V_1_	V_1_ Group	SL	SF	CT
1	0.016	Maintainer	− 0.0239 (↓)	0.036946 (↑)	− 0.00113 (↔)
2	− 0.084	Decliner	0.009167 (↑)	− 0.03152 (↔)	− 0.0008 (↔)
3	0.042	Improver	0.0434 (↑)	− 0.05609 (↓)	− 0.0007 (↔)
4	0.024	Maintainer	− 0.0058 (↔)	0.042065 (↑)	0.001033 (↔)
5	0.035	Improver	0.038967 (↑)	− 0.05788 (↓)	− 0.00248 (↓)
6	− 0.192	Decliner	0.0201 (↑)	− 0.09261 (↓)	0.0065 (↑)
7	− 0.136	Decliner	0.011 (↑)	0.002405 (↔)	0.0045 (↑)
8	− 0.204	Decliner	− 0.0046 (↔)	0.078085 (↑)	0.006725 (↑)
9	0.022	Maintainer	0.009067 (↑)	0.058508 (↑)	− 0.0067 (↓)
10	0.132	Improver	0.0359 (↑)	− 0.24397 (↓)	0.000975 (↔)
11	0.067	Improver	0.000 (↔)	0.016514 (↔)	0.00575 (↑)

V_1_, Last 5 m Velocity; SL, Step length; SF, Step frequency; CT, Contact time; 1–11, Athlete’s ID. Slopes represent the unstandardized linear regression coefficient calculated using the ordinary least-squares method across the four sequential attempts (A_1_ = 1, A_2_ = 2, A_3_ = 3 and A_4_ = 4). Individual response classifications are derived using literature-established analytical thresholds based on one-third of the typical error (TE): ±0.030 m/s/attempt for velocity (V_1_), ± 0.007 m/attempt for step length (SL), ± 0.033 steps/s/attempt for step frequency (SF), and ± 0.002 s/attempt for contact time (CT). (↑) indicates an increasing trajectory (Improver), (↓) indicates a decreasing trajectory (Decliner), and (↔) indicates a stable trajectory within the TE bounds (Maintainer). These classifications are strictly descriptive and exploratory. For inferential subgroup analyses, the three-category V1 classification was collapsed into a binary grouping: Velocity-Stable (Improvers and Maintainers combined; slope ≥ 0, *n* = 7) and Velocity-Declining (Decliners; slope < 0, *n* = 4). SL, SF, and CT individual classifications are retained as descriptive indicators of kinematic compensation strategies and do not constitute independent grouping criteria.

In the V_2_ model, neither BL (*β* = 0.111, 95% CI −0.091–0.314, *p* = .268) nor HR (*β* = 0.014, 95% CI −0.002–0.032, *p* = .084) showed a significant association with approach velocity (Tables 2 and 6; *R²m* = 0.222, *R²c* = 0.222). The equality of marginal and conditional R^2^ in this model suggests that inter-individual differences contributed negligibly to explained variance—a pattern strikingly distinct from the V_1_ model, in which random effects accounted for the vast majority of variance (*R²c* = 0.908).

In the V_3_ model, none of the step kinematic covariates showed a significant association with approach velocity (Tables 2 and 6; *R²m* = 0.074, *R²c* = 0.923): SL (*β* = 0.732, 95% CI −1.037–2.502, *p* = .403), CT (*β* = −2.868, 95% CI −12.698–6.963, *p* = .554), and SF (*β* = −0.208, 95% CI −0.686–0.270, *p* = .379). Exploratory testing was additionally performed with each covariate entered individually and in all binary and ternary combinations; as no significant association emerged in any specification (all *p* > .05), results are reported for the full three-covariate model.

**Table 6:** Fixed effects estimates for the exploratory linear mixed-effects models predicting last 5-meter approach velocity.

V_2_ predictor	β (m s^−1^ per unit)	95% CI	*p*
BL	0.1118	− 0.09101 to 0.3146	0.268
HR	0.0149	− 0.00217 to 0.0321	0.084

V_3_ predictor	*β* (m s^−1^ per unit)	95% CI	*p*
SL	0.7324	− 1.037 to 2.5020	0.403
CT	− 2.8679	− 12.698 to 6.9625	0.554
SF	− 0.2082	-0.686 to 0.2695	0.379

CI, Confidence interval; BL, Blood lactate; HR, Heart rate; SL, Step length; CT, Contact time; SF, Step frequency; V_2_, Exploratory model 1 (physiological marker model); V_3_, Exploratory model 2 (spatiotemporal step kinematics model). *β* coefficients represent unstandardized estimates computed via restricted maximum likelihood (REML). Due to the immediate nature of acute RPE, it was excluded from the *V*_2_ to preserve temporal homogeneity in post-effort monitoring.

**Table 7:** Pearson correlation analyses for individual V_1_ slope and internal load change indices (*N* = 11).

	V_1_ slope - ΔBL	V_1_ slope - ΔHR	V_1_ slope - ΔRPE	ΔHR–ΔBL
Pearson’s *r*	− 0.199	− 0.407	0.456	0.669*
p-value	0.558	0.214	0.159	0.024

Note. *n* = 11. Values represent Pearson r and p coefficients. V_*1*_ Slope = individual linear regression slope of last 5-metre approach velocity across four attempts; ΔBL = A4_(3)_ − A1_(3)_ blood lactate change (mmol·L⁻¹); ΔHR = A4_(3)_ − A1_(3)_ heart rate change (bpm); ΔRPE = A4_(0)_ − A1_(0)_ immediately post-effort rating of perceived exertion change (CR-10). All variables met normality assumptions (Shapiro–Wilk *p* > .05).

Pearson correlation analyses were conducted to examine associations between individual V_1_ slope and the magnitude of physiological load change across attempts. Results are presented in Table 7. No significant association was observed between V_1_ slope and ΔBL (*r* = − .199, *p* = .558), ΔHR (*r* = − .407, *p* = .214), or ΔRPE (*r* = .456, *p* = .159) (*n* = 11). Notably, a significant positive correlation was observed between ΔBL and ΔHR (*r* = .669, *p* = .024).

**Table 8:** Phase × group and attempt × group interaction model results for the full sample (*n* = 11).

	Model	F(df1, df2)	*p*	Notable contrasts
BL	Phase×group	F(8, 72) = 0.28	0.967	–
HR_post_	Phase×group	*F(8*,* 72) =* 0.23	0.984	–
RPE	Phase×group	*F(8*,* 72) =* 1.74	0.103	–
SL	Attempt×group	*F(3*,* 27) = 1.61*	0.209	–
SF	Attempt×group	*F(3*,* 27) = 1.24*	0.312	–
CT	Attempt×group	*F(3*,* 27) =* 2.24	0.106	–
V_1_	Attempt×group	*F(3*,* 27) =* 11.8	< 0.001	(A_3_ − A_1_) × (2 − 1): β = −0.50, *p* < .001 (A_4_ − A_1_) × (2 − 1): β = −0.58, *p* < .001
V_2_	Attempt×group	*F(3*,* 25) =* 0.36	0.778	–
**V**_**3**_	Attempt×group	*F(3*,* 24) =* 9.28	< 0.001	(A_3_ – A_1_) × (2 − 1): β=−0.45, *p*=.002; (A_4_ – A_1_) × (2 − 1): β=−0.57, *p*<.001

Subgroup classification was based on V_1_ slope: Velocity-Stable, Improvers + Maintainers (slope ≥ 0, *n* = 7); Velocity-Declining, Decliners (slope < 0, *n* = 4). This binary classification was applied consistently across all parameters. F = omnibus fixed effect test statistic; BL , Blood Lactate; HR, Heart rate; RPE, Rating of perceived exertion; SL, Step length; SF, Step frequency; CT, Contact time; V_1_, Last 5-m approach velocity; V_2_, Physiological marker model; V_3_, Spatiotemporal kinematic model. Notable contrasts reflect significant individual Phase × Group or Attempt × Group parameter estimates. (2 − 1) denotes the contrast between Velocity-Declining (Group 2) and Velocity-Stable (Group 1); negative β indicates lower values in the Velocity-Declining relative to the Velocity-Stable group. All models include a random intercept for Athlete_ID.

Phase × Group and Attempt × Group interaction model results are presented in Table 8. Interaction terms were non-significant for blood lactate (*F(8*,*72)* = 0.289, *p* = .967), RPE (*F(8*,*72)* = 1.74, *p* = .103), heart rate (*F(8*,*72)* = 0.230, *p* = .984), step length (*F(3*,*27)* = 1.61, *p* = .209), step frequency (*F(3*,*27)* = 1.24, *p* = .312), and contact time (*F(3*,*27)* = 2.24, *p* = .106).

The V_1_ model revealed a significant Attempt × Group interaction (*F(3*,*27)* = 11.80, *p* < .001), with significantly greater velocity decrements in the Velocity-Declining group at A_3_ (*β* = −0.50, *p* < .001) and A_4_ (*β* = −0.58, *p* < .001) relative to A_1_. The V_3_ model similarly yielded a significant interaction (*F(3*,*24)* = 9.28, *p* < .001); step length, step frequency, and contact time showed no significant fixed effects, yet between-group velocity divergence persisted at A_3_ (*β* = −0.45, *p* = .002) and A_4_ (*β* = −0.57, *p* < .001).

### Composite load index

**Fig. 10:**
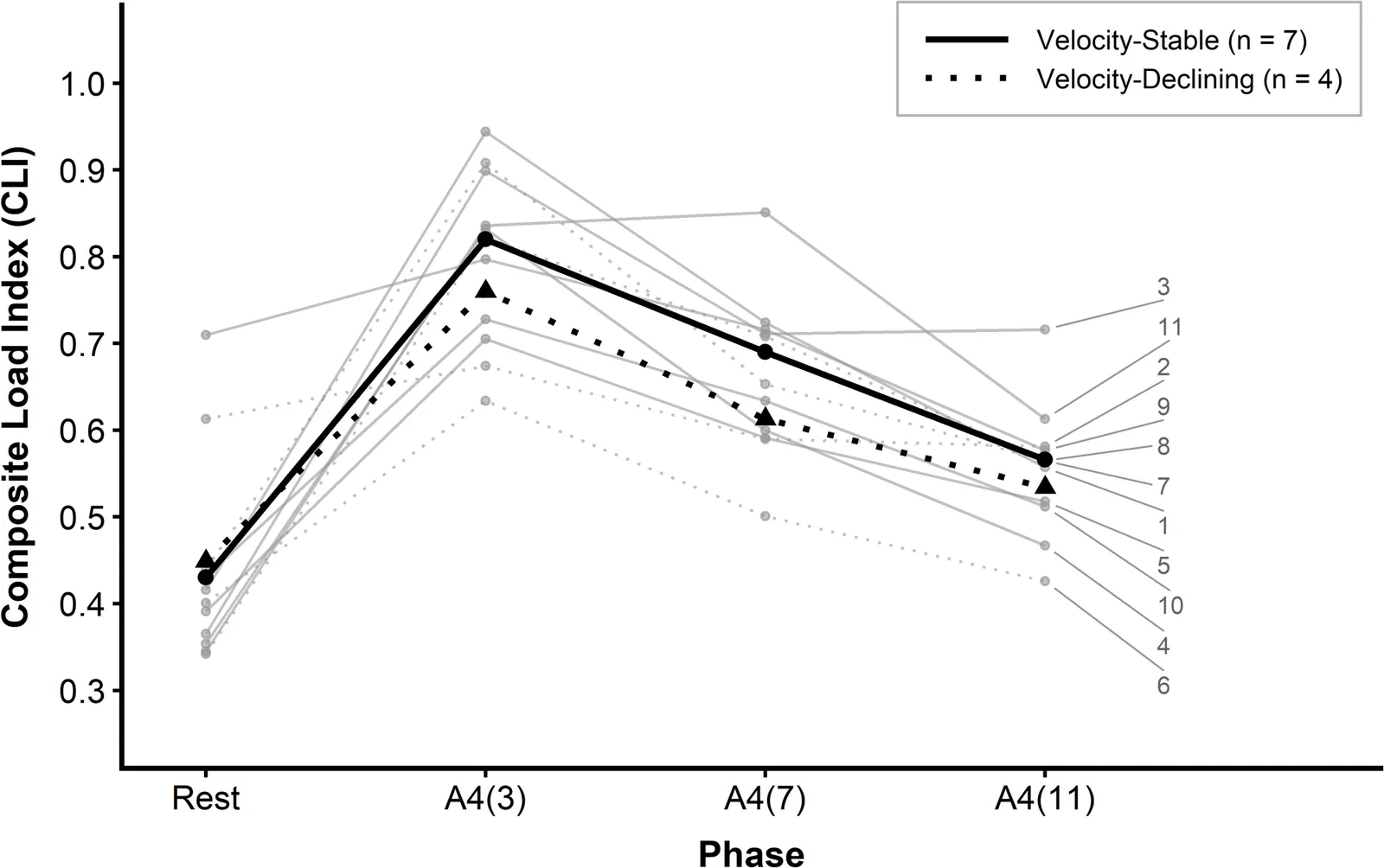
Composite load index (CLI) recovery kinetics across post-effort phases by velocity subgroup (Thin solid lines represent individual Velocity-Stable athlete trajectories (*n* = 7); thin dotted lines represent individual Velocity-declining athlete trajectories (*n* = 4). Numbers denote individual athlete identifiers. CLI = 0.42 × (HR/HR_peak_) + 0.30 × (BL/BL_peak_) + 0.28 × (RPE/RPE_peak_); all indicators normalised to individual peak values observed within the protocol. A_4(3)_, A_4(7)_, and A_4(11)_ denote 3, 7, and 11 min post-final attempt, respectively).

As a preliminary step toward individualised internal load monitoring, a Composite Load Index was computed for each athlete across the post-effort recovery phases (Rest, A_4(3)_, A_4(7)_, A_4(11)_). CLI values reached their peak at A_4(3)_ (Velocity-Stable: M = 0.82; Velocity-Declining: M = 0.76) and declined progressively through A_4(7)_ and A_4(11)_ in both subgroups (Fig. 10). Individual trajectories demonstrated considerable inter-athlete variability in both the magnitude of peak CLI and the rate of subsequent recovery.

## Discussion

The principal finding of this applied field-based study was that approach velocity remained statistically unchanged across four consecutive vault attempts performed below personal best height, despite increases in perceived exertion and moderate metabolic disruption. Regarding recovery kinetics, while BL and RPE returned to resting levels by the 11th minute of passive recovery, HR remained significantly above resting values at the same time point, reaching only warm-up levels. In the exploratory models, neither physiological indicators nor step parameters showed a significant association with approach velocity, and the large discrepancy between marginal and conditional R² values indicated that inter-individual differences, rather than phase effects, accounted for the majority of explained variance.

### Physiological and perceptual responses

At the group level, BL and RPE showed an increase from resting values to vault attempts; HR increased with warm-up and remained high throughout the protocol (Table 3). BL and HR showed no significant change between consecutive attempts, whereas RPE increased significantly between A_3_ and A_4_ () *p*_*Tukey*_ = 0.030; Table 3)

The fact that BL remained relatively stable for 4 repetitions is consistent with the repetitive high-intensity interval training structure of pole vault^6,7^. Phosphagen dominance may have limited metabolic accumulation during short explosive efforts, while the inter-attempt rest period (~ 3.5–4 min) allowed for partial phosphagen replenishment and prevented the accumulation of glycolytic stress^22^. This interpretation is consistent with evidence that approximately 70–90% of phosphocreatine stores can be replenished within 3–5 min in trained athletes, though PCr was not measured in the study^22^. The absence of progressive lactate accumulation also suggests that the protocol conditions may not have generated sufficient metabolic stress to drive further accumulation in this high-performance sample. Furthermore, BL values at the first attempt did not differ significantly from the run-up phase despite the 4–6 min rest period (Mean diff: − 0.18 mmol L^−1^, *p* = .999). Several explanations may account for this. Athletes may consciously regulate their approach velocity during attempts, knowing that a complex take-off and bar clearance technique must follow. This may possibly prevent them from reaching the velocity they would achieve with pole carrying alone, and this may have been reflected in their lactate response. Additionally, the elastic energy stored and released during pole bending and recoil may inherently limit the metabolic cost per attempt, while neuromuscular coordination during take-off may optimize kinetic energy to the pole, reducing additional muscle work^1,2,23^. These factors lie outside the scope of present study and should be treated as hypotheses for future research. Finally, the BL values measured in this study (A_4(₃)_ estimate ~ 3.62 mmol L^−1^) are consistent with the protocol conditions using half approach, 4 attempts, and a bar height set below personal best. Beaulieu et al. (1995) reported BL values between 4.22 and 7.74 mmol L^−1^ during pole vault in a decathlon competition^24^. These higher values likely reflect longer approach distances, a greater number of disciplines, and higher overall competition stress. Reference BL data specific to pole vault competition conditions remain scarce; the values obtained in the present study may therefore offer a preliminary contextual benchmark for future comparisons.

HR increased from the warm-up phase and remained at a relatively similar level throughout successive attempts. The collection of HR data at 3 min post-effort suggests that sympathetic activation associated with each effort may have partially subsided by the next measurement point, which may have contributed to the absence of significant HR differences between consecutive attempts. The relatively stable HR across attempts may further reflect habituation to repeated stimuli and progressive parasympathetic reactivation between efforts^25,26^. Although statistical significance was not reached, the numerically highest HR value was observed at A_4(3)_ as a point estimate, which may suggest that cumulative sympathetic load increased slightly toward the final attempt.

Unlike HR and BL, the continued and significant increase in RPE after the 3rd attempt is consistent with the interpretation that RPE reflects an integrated dimension encompassing multiple afferent and central signals^27,28^. The psychobiological model suggests perceived effort reflects conscious awareness of the motor command rather than peripheral afferent feedback^29,30^. Within this framework as trials accumulated, motor activation may have increased to maintain approach velocity without generating sufficient cardiovascular or metabolic signals. This interpretation aligns with the central governor model^31^, which suggests that central fatigue does not necessarily progress simultaneously with peripheral metabolic accumulation; however, this interpretation remains speculative, as our data do not include direct measures of motor command magnitude or neural drive. Additionally, athletes’ individual assessments of demand-coping processes may have further modulated RPE independently of objective physiological state^32^. Furthermore, it has been reported that knowledge of the task endpoint can affect perceptual and performance kinetics^33^, suggesting that an athlete’s awareness of the total number of attempts may shape both their RPE pattern and pacing strategy. The impact of such knowledge on motor planning and effort perception in pole vault warrants examination in future research.

When recovery kinetics were examined, notable differences were observed among the parameters. BL returned to values comparable to warm-up levels at 7 min after the final attempt (WU_3_ vs. A_4(7)_: *p*_*Tukey*_ = 0.781), and both BL (R vs. A_4(11)_: *p*_*Tukey*_ = 0.375) and RPE (R vs. A_4(11)_: *p*_*Tukey*_ = 0.894) reached values similar to resting levels by 11 min. The relatively earlier return of lactate to baseline is consistent with the enhanced metabolite removal capacity associated with greater mitochondrial density and intercellular lactate shuttle activity in trained athletes^34^. In contrast, HR at the end of 11 min of passive recovery had reached warm-up levels (*p*_*Tukey*_ = 0.327) but remained significantly above resting values (*p*_*Tukey*_ = 0.001). These differing recovery rates highlight that the three indicators reflect physiological mechanisms that do not completely overlap. Blood lactate is primarily cleared via oxidative phosphorylation and gluconeogenesis, while HR is dependent on autonomic regulation, parasympathetic reactivation, and sympathetic withdrawal^35^. RPE, on the other hand, is defined as a composite indicator encompassing multiple afferent and central signaling sources^27,28^. In this context, relying on a single indicator in training management may prove insufficient.

### Approach velocity and kinematic parameters

At the group level, none of the approach velocity and step parameters showed significant change over the four attempts (Table 2; **V**^1^: *p* = .334, **SL**: *p* = .232, **SF**: *p* = .699, **CT**: *p* = .432). In exploratory models, neither physiological indicators (HR, BL, RPE) nor step parameters (SL, SF, CT) showed a significant relationship with approach velocity (Table 6). The findings of the V_2_ model should be interpreted with this mismatch in mind: approach velocity was analyzed during the attempt, whereas blood lactate and heart rate were collected post-effort. This limits the interpretability of load-velocity relationships and means that the V_2_ model reflects recovery-phase physiological status rather than concurrent within-attempt load. The equality of R^2^m and R^2^c in the V2 model (0.222) is notably distinct from the pattern observed in the V1 model, where random effects accounted for the vast majority of explained variance (R^2^c = 0.908). This suggests that, unlike approach velocity, post-effort HR and BL values showed relatively little inter-individual variability in their relationship with velocity—that is, the between-athlete differences that dominated the velocity model did not manifest in the physiological covariates in the same way. One possible explanation is that post-effort HR and BL responses at the 3rd minute were similarly distributed across athletes under these controlled preparatory-phase conditions, leaving little variance for the random intercept to explain beyond the fixed effects. However, given the small sample size and the exploratory nature of this model, this pattern should be interpreted cautiously and warrants replication in larger samples. With this constraint in mind, this three-layered null finding, considered as a whole, suggests that velocity generation under the controlled conditions studied is maintained relatively independently of both physiological load and kinematic parameters. This is similar to the idea that highly automated running motor programs in trained pole vaulters function relatively unaffected by peripheral fatigue signals^36^. Compared to studies on running biomechanics where changes in kinematic parameters were observed along with muscular fatigue^37^, it is noteworthy that no such changes were observed in the present study. However, whether this difference stems from fatigue resistance or insufficient stimulus is addressed in the later limitations section.

Given the critical importance of approach run kinematics for performance in pole vault^19,38,39^, their preservation may indicate that technical stability can be maintained despite physiological load. Schade et al. reported that world champion Blom maintained a high level of approach velocity (9.03–9.04 m/s) in four successful vaults at the 2005 IAAF World Championships final^39^. This finding demonstrates that velocity conservation is possible in highly trained pole vaulters despite competition load; however, it should be noted that this study is a case study of a single athlete, and it remains debatable whether the present protocol generated sufficient fatigue stimulus to test these mechanisms. It is also known that low velocity variability is a well-established characteristic in elite pole vaulters, supporting the conservation of velocity and step kinematics^40,41^. Walsh and Hall emphasized the critical importance of velocity stability in the final meters of the approach run^41^, while Cassirame et al. revealed kinetic-kinematic differences between successful and unsuccessful attempts^42^. These studies support the view that the motor pattern may reflect a highly conserved motor program rather than adaptive changes. In this context, the conservation of both velocity and step parameters at the group level in our study is particularly noteworthy. Although Makaruk et al. described step length and step frequency as a dual regulation mechanism that compensates for each other^43^, it is important to note that no such obvious regulation was observed in the present study. None of the athletes who maintained, improved, or decreased their velocity exhibited a consistent compensation mechanism at this level; rather, each performed entirely unique kinematic adjustments (Table 5).

### Individual heterogeneity

One of the key findings of this study is the significant individual differences masked by the group mean analysis. According to the slope-based classification, athletes were divided into three groups in terms of velocity: Maintainer (27.3%), Decliner (36.4%), and Improver (36.4%). The equal size of the Decliner and Improver groups may have led to the statistical balancing of opposing individual tendencies in the group mean. This pattern is consistent with the significant difference between the marginal and conditional R² in the velocity model (*R²m* = 0.004, *R²c* = 0.908). The vast majority of the explained variance stems from inter-individual differences, not phase effects.

Individual heterogeneity at the kinematic level exhibited similar complexity. While 63.6% of athletes were classified as Improver in terms of step length, this figure remained at 36.4% in terms of velocity. This suggests that an increase in SL does not always translate into an increase in velocity. Remarkably, some athletes in the Decliner group experienced a loss of velocity while increasing their step length (Table 5). These heterogeneous kinematic patterns may explain why exploratory models fail to identify a significant group-level relationship between step parameters and velocity: different athletes may have followed entirely different kinematic regulation strategies, which may have statistically cancelled each other at the population mean. This is consistent with findings in the sprint literature examining repetitive effort trajectories. Hureau et al. and Billaut et al. provided evidence suggesting that in repetitive sprint protocols, mechanical performance trajectories are individually modulated by the central motor drive according to athlete-specific peripheral fatigue thresholds^33,44^. These variations suggest that athletes may develop individualized fatigue responses that are either step-length-dependent or step-frequency-dependent, depending on their neuromuscular capacities. Therefore, slope-based individual trajectory analysis may offer coaches a complementary monitoring approach to identify athlete-specific kinematic adaptations across repeated efforts, beyond what group-level statistics can reveal.

This pattern of individual heterogeneity is statistically supported by the subgroup interaction analyses (Table 8). The non-significant Phase × Group interaction terms for blood lactate, heart rate, and step kinematics indicate that trajectories in these parameters did not differ systematically between Velocity-Stable and Velocity-Declining athletes, suggesting that velocity loss is not a direct reflection of physiological load accumulation or kinematic adjustment.

However, the Attempt × Group interaction for approach velocity was significant in both the V_1_ and V_3_ models. Although kinematic covariates showed no significant fixed effects in the V_3_ model, between-group velocity divergence persisted, indicating that velocity loss in the Velocity-Declining group cannot be explained by changes in step length, step frequency, or contact time. This pattern is consistent with the motor program hypothesis and suggests that neuromuscular or central fatigue mechanisms may underlie individual velocity response variation^36^. However, this interpretation remains speculative in the absence of direct EMG or motor unit activation data.

Pearson correlation analyses revealed no significant association between individual V_1_ slope and ΔBL, ΔHR, or ΔRPE, indicating that heterogeneity in individual velocity trajectories cannot be systematically explained by differential physiological load accumulation (Table 7). The significant positive correlation between ΔBL and ΔHR confirms the internal consistency of metabolic and cardiovascular load indicators across individuals.

### Practical implications

These findings offer several practical implications for pole vault training monitoring.

First, monitoring individual velocity and kinematic trajectories in addition to group averages can enable early detection of individual velocity losses that might be masked by the group-level mean. Indeed, in this study, the group-level average concealed the fact that some athletes experienced meaningful velocity loss across attempts. The subgroup interaction analyses further demonstrated that velocity loss in the Velocity-Declining group was not accompanied by systematic changes in metabolic load or step kinematics, suggesting that standard physiological monitoring alone may be insufficient to identify athletes at risk of velocity decline.

Second, the differential recovery kinetics observed across indicators have direct implications for training planning. Blood lactate and RPE returned to resting levels within 11 min of passive recovery, while HR remained significantly elevated at the same time point. Recovery decisions based on a single indicator may therefore be misleading; multidimensional monitoring is recommended. To support this, a preliminary Composite Load Index (CLI) was developed, integrating HR, BL, and RPE weighted by their empirically observed recovery rates (HR: w = 0.42; BL: w = 0.30; RPE: w = 0.28), normalised to each athlete’s individual peak values. CLI reached its peak at A4(3) and declined progressively through A4(11), providing a single integrated metric for tracking cumulative load and recovery status across repeated vault attempts. The CLI is proposed as an exploratory monitoring tool; prospective validation in larger samples is required before applied use.

Third, the finding of kinematic heterogeneity has applied importance for individualised training design. The observation that some athletes lost velocity while simultaneously increasing step length highlights the importance of monitoring athlete-specific kinematic strategy changes and providing individual-level feedback, rather than relying solely on group-level kinematic norms.

Finally, future research should employ protocol designs that more closely reflect high-fatigue competition conditions — including shorter rest intervals, full approach distances, progressive bar height increases, and a greater number of attempts. Findings from such protocols will allow testing whether the kinematic and velocity conservation observed under the controlled conditions of the present study can be generalised to real competitive stress.

### Limitations

Several important methodological limitations must be considered in this study.

First, the protocol may not have generated sufficient fatigue stimulus to challenge velocity or step kinematics, considering the half-approach distance (~ 18 m), four attempts, and relatively long rest intervals (~ 3.5–4 min). While bar height was set below personal best to ensure successful clearance and eliminate psychological performance anxiety rather than to reduce physiological demand, the absence of a matched maximal-effort control condition means that a ceiling effect cannot be fully excluded. The observed velocity and kinematic conservation may therefore reflect insufficient stimulus rather than true fatigue resistance. Full competition conditions—such as progressively increasing bar height, rest intervals that may be shorter in consecutive attempts, full approach distance, and competitive pressure—are likely to create greater challenges to velocity conservation. Short recovery intervals have been shown to restrict phosphagen regeneration and increase reliance on glycolytic metabolism^6,22^. Similarly, increasing bar height can increase both perceptual difficulty and psychological pressure, which may enhance sympathetic activation and restrict inter-vault recovery or increase the perceived cost of effort^26^. Full approach distance may further increase glycolytic contribution and metabolite accumulation^45^. Given this theoretical framework, it is possible that the present protocol fell below the threshold required to challenge approach velocity.

Second, the small and non-homogeneous sample size (*n* = 11) limits the generalizability of the findings. The sample size is consistent with the literature^3,15^. Nevertheless, small effects may have gone undetected in the analyses (power calculations indicated 80% power to detect medium effects, f = 0.30). Exploratory models were run under conditions where statistical power may be insufficient; therefore, null findings should not be considered definitive proof of the independence between physiological load and kinematic parameters and approach velocity, but rather a set of hypotheses for larger sample studies to test these relationships. The participants’ high training level (six of whom compete at the national team level) limits the generalizability of the results to broader populations. Additionally, the imbalanced sex distribution in the study (4 women, 7 men) made sex-specific analyses impossible. Although the bar height in the protocol is adjusted for each athlete, the wide personal-best range may also have introduced heterogeneity in the relative task difficulty.

Third, bar clearance success was not recorded as a formal outcome variable; however, all attempts resulted in successful clearance by protocol design, as bar height was set to ensure this. Whether kinematic conservation observed under these conditions would translate to higher-stakes competitive attempts—where clearance success is not guaranteed—remains an open question for future research. Relatedly, approach velocity was derived from 2D rather than 3D video analysis, which precluded the evaluation of out-of-plane movements and may have introduced minor positional estimation errors. Intra-rater reliability checks were conducted with a one-day interval between sessions; this may have inflated the ICC estimate, and the actual measurement error may be slightly larger than reported.

Fourth, the fixed recovery interval employed in the present study does not permit retrospective simulation of variable recovery groupings. Real pole vault competition involves highly variable inter-attempt rest periods depending on the number of competitors, session duration, and bar progression dynamics. A dedicated variable-interval protocol—incorporating rest periods ranging from under 5 min to over 30 min—would enable individualised recovery threshold modelling for Velocity-Stable and Velocity-Declining subgroups and is proposed as a key direction for future research.

Lastly, the central mechanistic question raised by this study—whether velocity maintenance reflects the sustained recruitment of motor units, the preservation of the stretch-shortening cycle function, or compensatory technical adjustments—cannot be answered without direct neuromuscular assessment. Future protocols should include surface EMG of gastrocnemius, quadriceps, and hip flexors to directly assess motor unit recruitment stability and compensatory neuromuscular adjustments across attempts—a mechanistic gap that the present study cannot address. Additionally, counter-movement jump height or force platform measurements alongside physiological and kinematic variables would help distinguish these pathways and more precisely characterize the mechanisms of fatigue resistance specific to pole vault. Furthermore, a matched maximal-effort control condition—incorporating vault attempts at athletes’ personal best heights under identical recovery intervals—would allow direct between-session comparison of velocity, blood lactate, heart rate, and step kinematics, enabling quantitative differentiation between genuine fatigue resistance and insufficient fatigue stimulus. Such a design would substantially strengthen the causal validity of the load-performance dissociation observed in the present study.

## Conclusion

The aim of this study was to examine the temporal changes in physiological load indicators during repeated pole vault attempts and to investigate the relationship between approach velocity and these physiological parameters and step kinematics at both group and individual levels.

A common assumption in athlete load management is that increased physiological load leads to impaired mechanical output. The present findings suggest that, despite progressive increases in perceived exertion and moderate metabolic disruption under the controlled conditions examined, no statistically significant change was observed in approach velocity or step kinematics across four repeated attempts. This pattern is consistent with the interpretation that the relationship between physiological load and technical performance is not necessarily linear, and that sports requiring explosive, equipment-dependent repetitive effort may possess mechanisms capable of maintaining mechanical output under the controlled conditions examined. Furthermore, recovery kinetics differed across indicators: while BL and RPE returned to resting levels within 11 min of passive recovery, heart rate reached only warm-up levels at the same time point, underscoring the importance of multidimensional load monitoring.

Individual trajectory analysis revealed meaningful heterogeneity in velocity and kinematic strategies beneath the stable group mean, suggesting that group-level null findings should be interpreted with caution.

To the authors’ knowledge, this study represents one of the first observations consistent with load-performance dissociation under submaximal conditions in a closed-skill, apparatus-dependent sport. However, since the present protocol may not have induced sufficient fatigue under maximal competitive demands to challenge approach velocity, the possibility of a ceiling effect cannot be ruled out. These results should therefore be interpreted not as evidence of fatigue resistance under maximal competitive demands, but rather as an indication of performance maintenance under the specific conditions examined.

## Data Availability

The data will be made available upon request.
